# Costly Inheritance and the Persistence of Insecticide Resistance in *Aedes aegypti* Populations

**DOI:** 10.1371/journal.pone.0123961

**Published:** 2015-05-01

**Authors:** Helio Schechtman, Max O. Souza

**Affiliations:** 1 Programa de Computação Científica, Fundação Oswaldo Cruz, Rio de Janeiro, RJ, Brasil; 2 Departamento de Matemática Aplicada, Universidade Federal Fluminense, R. Mário Santos Braga, Niterói, RJ, Brasil; University of Queensland & CSIRO Biosecurity Flagship, AUSTRALIA

## Abstract

Global emergence of arboviruses is a growing public health concern, since most of these diseases have no vaccine or prevention treatment available. In this scenario, vector control through the use of chemical insecticides is one of the most important prevention tools. Nevertheless, their effectiveness has been increasingly compromised by the development of strong resistance observed in field populations, even in spite of fitness costs usually associated to resistance. Using a stage-structured deterministic model parametrised for the *Aedes aegypti*—the main vector for dengue—we investigated the persistence of resistance by studying the time for a population which displays resistance to insecticide to revert to a susceptible population. By means of a comprehensive series of in-silico experiments, we studied this reversal time as a function of fitness costs and the initial presence of the resistance allele in the population. The resulting map provides both a guiding and a surveillance tool for public health officers to address the resistance situation of field populations. Application to field data from Brazil indicates that reversal can take, in some cases, decades even if fitness costs are not small. As by-products of this investigation, we were able to fit very simple formulas to the reversal times as a function of either cost or initial presence of the resistance allele. In addition, the in-silico experiments also showed that density dependent regulation plays an important role in the dynamics, slowing down the reversal process.

## Introduction

Arthropods vectors are at the crossroads of current human development as health authorities are observing a global re-emergence of many vector-borne diseases such as malaria, dengue and chikungunya disease [1–5], probably due to the fast spread of some of the associated vectors in many areas of the world [6, 7]. Vector-borne diseases can have a wide spectrum of morbidity varying from mainly benign to lethal, and also have a large impact on child mortality—e.g. severe dengue is a leading cause of serious illness and death among children in Asian and Latin American countries [1]. Recurring epidemics of these vector-borne diseases are likely to cause a large burden to national health systems [8, 9], even if these diseases were solely benign. However, even typically more innocuous diseases seem to be undergoing transformations and are evolving towards more severe forms [10, 11].

For a considerable number of diseases associated to arboviruses, there is neither aetiological treatment nor chemoprophylaxis [1, 8]. In addition, there is no licensed vaccine available for them, even though several candidate vaccines are currently being evaluated in clinical studies [12–14]. In this scenario, control of the vector population is possibly the best alternative available to health authorities for disease prevention, and lack of such an adequate control might lead to periodical occurrence of epidemics—cf. [15–18].

Control of these arthropod vectors is particularly important in areas where they are widespread, and to a large extent is achieved by use of chemical insecticides that target them at a particular stage of their life-cycle, such as larvicides or adulticides [19]. Organophosphorus and carbamate insecticides affect nerve synapses through the neuro-transmitter acetylcholinesterase, while the organochlorines (DDT) and pyrethroids aim the sodium channels of the nerve sheath, producing an effect similar to a knockdown.

Many of the pyrethroid insecticides are applied by public health officials and trained personnel as an ultra low-volume (ULV) spray, which stays in the air and kills adult mosquitoes on contact. However, not only are pyrethroid-derived insecticides extensively used in agriculture, but they are also found in many commercial products available on store shelves, such as household insecticides, pet sprays and shampoos, lice treatments, mosquito repellents and impregnated bed nets, as mentioned in [20].

Arthropod vectors are becoming increasingly more resistant to those insecticides [21–25] probably due to their intensive, and sometimes indiscriminate, use. Insecticide resistance might undermine a number of control programs that exist worldwide [20], and therefore has received considerable attention by regional and worldwide health organizations: a number of protocols were developed by these agencies to identify and manage such behaviour [26].

There are several mechanisms that might lead to development of insecticide resistance, which broadly fall in four categories: metabolic, target-site alteration, behavioural, and penetration resistance [20, 27, 28]. In all cases, resistance is likely to be genetically mediated, and due to mutations in one or more genes [29, 30]. Specifically, in the case of pyrethroid-based insecticides the mechanism for resistance is target-site alteration, *i.e.* a genetic mutation also known as Knock-Down Resistance—*kdr*.

In *Aedes aegypti*, these *kdr* genes have been mainly identified, although there is some controversy as to the question of single or multiple genes being necessary for resistance development [27, 31–33]. Once a mutation occurs it can spread very fast, as it can be observed in the case of *Aedes aegypti* for which there are reports of populations where the frequency of the mutant allele 1534Cys is above 95%, and there is also evidence of slow reversal in the absence of insecticide pressure [34].

In the particular case of the anthropophilic *Aedes aegypti*, the Arthropod Pesticide Resistance Database [35] registers almost four hundred cases of resistance to twenty nine active substances, in almost two hundred locations around the world. A recent picture of the situation of *Aedes aegypti* insecticide resistance in Brazil shows a geographical distribution that ranges from almost no resistant insects up to fixation of the resistant variety [36].

Insecticide resistance usually comes with associated fitness costs, and these have a large variance ranging from nearly absent to very high costs [27, 28, 30, 37, 38]. In the former case, fixation of the resistant variety is likely [29, 30, 39], while in the latter it might lead to a very resistant vector that is incapable of transmitting the disease [40] and/or, alternatively, removal of the selective pressure might greatly decrease the frequency of the resistance associated alleles after some time. Such a large variance seems to be due not only to the several mechanisms operating, but also to the fact that a number of different issues affects its development and evolution, and hence can lead to a quite complex evolutionary architecture—cf. [41].

The question of how resistance costs impinge on life parameters is a large and controversial one. Usually resistance costs are reported in association with reduced lifespan and oviposition rates; sometimes also longer maturation times [42]. On the other hand, correlation of larger transition times with fitness advantage for resistant strains has also been found—cf. [22] and references therein. For instance [43] studied four field resistant populations in Brazil and observed a variety of scenarios. This included negligible overall variation in maturation times, thus suggesting a negligible fitness cost in this case; for some other populations, the changes were overall large.

With this picture in mind, we employed an *in silico* model adapted from [18], and parametrised it for *Aedes aegypti*—which is a highly competent vector for dengue and the most important one [1]. We then investigated the quantitative and qualitative characteristics of the dynamics of mosquito populations with resistant individuals in the absence of selective pressure for an extensive range of the resistance allele initial frequency and fitness costs.

## Methods

### Mathematical model

**Fig 1:**
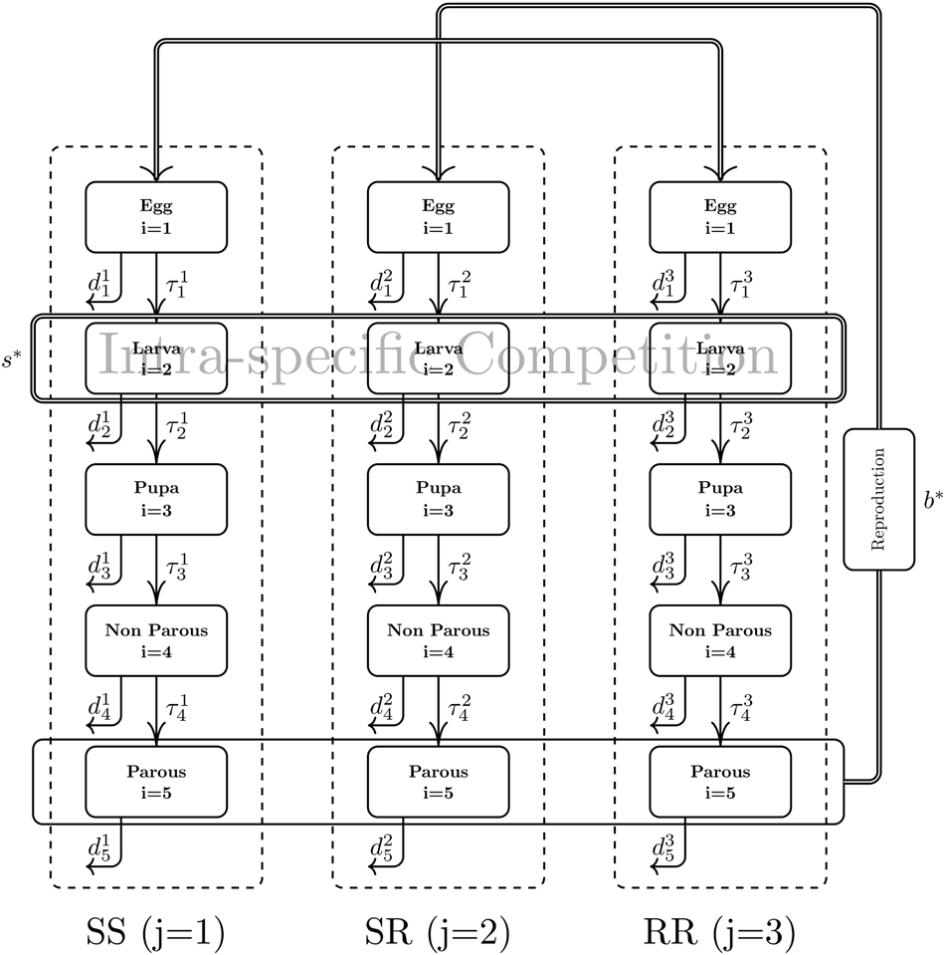
Compartmental model representation. The five life-stages of susceptible (SS), heterozygous (SR) and resistant (RR) mosquitoes are represented by rectangular boxes which are arranged in columns according to its genotype. Arrows connecting two boxes represents mosquitoes of a specific life stage maturing into another life stage, with the appropriate transition rate. Outward arrows represent removal of individuals from that specific life stage due to natural death. The thicker trifurcated arrow connecting the Parous stage to the Egg stage boxes represents an influx of eggs arising from random, non-preferential mating of parous winged mosquitoes. The *Intra-specific Competition* box represents a logistic type competition among larvae. Parameters are given in Table 1.

We describe the population dynamics of *Aedes aegypti* with a stage-structured model comprising five stages, and three genotypes that are associated to a genetically mediated resistance by a single locus with two alleles. A pictorial description of the model is given in Fig 1 while the description of the parameters—together with their assumed baseline values—is presented in Table 1.

**Table 1:** Model parameters.

Parameter	Value	Definition
$d_{1}^{1}$	0.01005	baseline egg death rate
${\bar{\tau }}_{1}^{1}$	1/120	egg to larva mean transition rate
$d_{2}^{1}$	0.10536	baseline larva death rate
${\bar{\tau }}_{2}^{1}$	1/12	larva to pupa mean transition rate
$d_{3}^{1}$	0.01005	baseline pupa death rate
${\bar{\tau }}_{3}^{1}$	1/2	pupa to non-parous winged mosquito mean transition rate
$d_{4}^{1}$	0.02020	baseline non-parous winged mosquito death rate
${\bar{\tau }}_{4}^{1}$	1/5	non-parous to parous winged mosquito transition rate
$d_{5}^{1}$	0.06187	baseline parous winged mosquito death rate
*b* ^(1,1)^	4	baseline oviposition rate
*s* ^1^	10^−6^	baseline per capita intra-specific death rate
*θ*_1_	1.8326	phase shift for egg to larva transition rate
*θ*_2_	1.0472	phase shift for larva to pupa transition rate
*θ*_3_	1.0472	phase shift for pupa to non-parous winged mosquito transition rate
*δ*	0.25	amplitude of periodic perturbation

Transition, death, oviposition rates, amplitude of periodic perturbation and phase shifts for susceptible individuals in the model depicted in Fig 1. Rates are given in *day* ^−1^, the *s* ^1^ parameter is given in *day* ^−1^ ⋅ *individual* ^−1^ whilst phase shifts are presented in radians. The parameter *δ* is dimensionless.

The stages of the model are indexed by a subscript *i* = 1, …, 5. Three of the five stages represent the immature forms—eggs, larvae, and pupae—which are indexed as *i* = 1, 2, 3, respectively. The other two represent the adult forms—non-parous, and parous winged mosquitoes—and are indexed as *i* = 4, 5, respectively.

The two resistance-mediating alleles are denoted by S, for the wild type, and R for the mutant resistant variety. The corresponding genotypes—SS, SR and RR—are indexed by a superscript *j* = 1, 2, 3, respectively.

One of the features of the model is the description of the costs incurred by the genotypes with the mutant allele. These costs, which may potentially affect several parameters within the model, are conveniently described by a **cost function** C, which takes two arguments: a genotype index, and a parameter symbol. For a given pair of arguments, its value is a **modulating cost**, *i.e.* it is the modulus of the relative change with respect to the baseline value in the given parameter (*e.g.* death rate), incurred by the given genotype (*e.g.* see Eq (1) below). The choice of a particular cost function reflects the modelling assumptions about the effects of resistance on the various life parameters, and it can also be thought of as a **cost structure**. Naturally, C makes sense only for genotypes with the mutant variety, *j* = 2, 3, but it is convenient to allow it to be defined for the wild genotype with a value of zero.

At each stage individuals are removed due to death. We assume constant death rates for each stage and genotype, and write the death rate corresponding to stage *i* and genotype *j* as $d_{i}^{j}$. In particular, $d_{i}^{1}$ is the baseline death rate of individuals of the wild genotype at stage *i*. For the other genotypes, we have

$$\begin{array}{c} d_{i}^{j}=d_{i}^{1}(1+C(j,d_{i}^{1})),\;j=2,3 \end{array}.$$ (1)

Individuals transit from stage *i* = 1, …, 4 to stage *i* + 1 at a rate $\tau_{i}^{j}(t)$. Individuals in stage *i* = 5—parous winged mosquitoes—can only exit that stage due to death. Individuals from stage *i* = 4—non-parous winged mosquitoes—transit to stage *i* = 5—parous winged mosquitoes—at a constant rate $\tau_{4}^{j}(t)={\bar{\tau }}_{4}^{j}$. The remaining stages—egg, pupae and larvae—have transition rates that are highly dependent on seasonal influence—mainly humidity and temperature. Therefore, they exhibit seasonality themselves, and hence are periodic in time [18, 44–47]. Following [18], we have taken these transition rates to be given by

$$\begin{array}{c} \tau_{i}^{j}\left(t\right)={\bar{\tau }}_{i}^{j}(1+\delta \sin(\frac{2\pi t}{\bar{y}}+\theta_{i})),\;i=1,2,3,\;j=1,2,3; \end{array}$$ (2)

where ${\bar{\tau }}_{i}^{j}$ is the corresponding mean rate, *δ* is the magnitude of the periodical perturbation, $\bar{y}=365.25$ is the number of days in a year, and *θ*_*i*_ are phase shifts. The mean transition rates were taken to be genotype independent, *i.e*., ${\bar{\tau }}_{i}^{j}={\bar{\tau }}_{i}^{1}$, *i* = 1, …, 4 and *j* = 2, 3. In the context of the cost function this amounts to choosing:

$$\begin{array}{c} {\bar{\tau }}_{i}^{j}={\bar{\tau }}_{i}^{1}(1-C(j,{\bar{\tau }}_{i}^{1})),\;C(j,{\bar{\tau }}_{i}^{1})=0,\;i=1,\ldots ,4,\;j=2,3. \end{array}$$ (3)

where ${\bar{\tau }}_{i}^{1}$ is the corresponding baseline rate.

The phase shifts in Eq (2) assume that *t* = 0 corresponds to 1st of January, in order to account for the influence of summer and rainy season in the Brazilian city of Rio de Janeiro. These phase shifts correspond to peak transitions rates at mid-December for $\tau_{1}^{j}$ (egg to larvae), and end of January for $\tau_{2}^{j}$ (larvae to pupae) and $\tau_{3}^{j}$ (pupae to non-parous winged mosquitoes), respectively. The magnitude of the periodic perturbation was chosen to be $\frac{1}{4}$ as in [18], and it approximately matches the seasonal amplitude variation of the Breteau larval infestation index as observed in the city of Rio de Janeiro from 1997 to 2003.

Eggs are laid by parous winged mosquitoes, under the assumptions of random mating and Mendelian inheritance. The baseline oviposition rate, *b* ^(1,1)^, is the average number of eggs laid per adult, per day, assuming both parents are of the genotype SS. The oviposition rates for the other two genotypes SR and RR—*b* ^(2,2)^ and *b* ^(3,3)^, respectively—are defined as the baseline rate *b* ^(1,1)^ penalised by the corresponding costs:

$$\begin{array}{c} b^{\left(j,j\right)}=b^{\left(1,1\right)}\left(1-C\left(j,b^{\left(1,1\right)}\right)\right),\;j=2,3. \end{array}$$ (4)

For mating encounters with parents of different genotypes, we use the geometric mean of the corresponding rates:

$$\begin{array}{c} b^{\left(m,n\right)}=\sqrt{b^{\left(m,m\right)}b^{\left(n,n\right)}},\;m,n=1,\ldots ,3,\;m\neq n. \end{array}$$ (5)

These rates are then used to define the oviposition function *B* that take account of the random sexual mating between males and females by genotype—see System (Eq 8) and further details in S1 Text.

We assume that larvae undergo intra-specific competition, which then leads to density dependent death. Such an intra-specific competition has indeed been observed in [48–50]. In the present model, the death rate arising from this competition is proportional to the total number of larvae in the population, with the corresponding proportionality coefficient denoted by *s* ^*j*^ and given by:

$$\begin{array}{c} s^{j}=s^{1}\left(1+C\left(j,s^{1}\right)\right),\;j=2,3. \end{array}$$ (6)

where *s* ^1^ is the baseline per capita intra-specific death rate. From an ecological point of view, this means that all genotypes exert identical competitive effect, but have different competitive responses.

Other underlying assumptions are large homogeneous populations, with random non-preferential mating, and a 1:1 sex ratio. Resistance was assumed, except when explicitly indicated otherwise, to impinge a uniform modulating cost on oviposition, death and per capita intra-specific death rates for all the genotypes that have the resistance allele R. This assumption on the resistance costs amounts to using the following cost function:

$$\begin{array}{c} C\left(j,\cdot \right)=\{\begin{array}{cc} 0, & j=1;\\ C, & j=2,3. \end{array} \end{array}$$ (7)

Therefore, from now on, whenever we refer to a fitness cost, or simply a cost, we mean the value of such uniform cost *C*.

The present model is a slight modification of the model in [18]. The most noticeable differences are the lack of an imposed mutation rate, the egg distribution given by Mendelian inheritance due to mating rather than using a gene-pool under Hardy-Weinberg equilibrium assumption, and the dependence of the per capita intra-specific death rate on the genotype.

Under the above assumptions, a natural choice is to describe the model using a set of ODE’s as follows:

$$\begin{array}{c} \left\{ \begin{array}{cc} {\dot{Y}}_{1,j} & =B\left(j,Y_{5,\cdot }\right)-(\tau_{i}^{j}\left(t\right)+d_{i}^{j})Y_{1,j},\\ {\dot{Y}}_{2,j} & =\tau_{i}^{j}\left(t\right)Y_{1,j}-(\tau_{i}^{j}\left(t\right)+d_{i}^{j}+s^{j}\sum_{l=1}^{3}Y_{2,l})Y_{2,j},\\ {\dot{Y}}_{3,j} & =\tau_{i}^{j}\left(t\right)Y_{2,j}-(\tau_{i}^{j}\left(t\right)+d_{i}^{j})Y_{3,j},\\ {\dot{Y}}_{4,j} & =\tau_{i}^{j}\left(t\right)Y_{3,j}-(\tau_{i}^{j}+d_{i}^{j})Y_{4,j},\\ {\dot{Y}}_{5,j} & =\tau_{i}^{j}Y_{4,j}-d_{i}^{j}Y_{5,j}; \end{array}\right. \end{array}$$ (8)

where *t* is measured in days, and *Y*_*i*,*j*_ denotes stage *i* within genotype *j* = 1, 2, 3. The oviposition function *B*(*j*, *Y*_5,⋅_) models the production of eggs of a specific genotype due to the sexual encounters of female and male parous winged mosquitoes, and is further detailed in the S1 Text.

The baseline oviposition, transition and death rates parameters used in the model were taken from [18], who tabulated the data found on various literature sources. The single exception was the baseline per-capita intra-specific death rate, *s* ^1^, which was taken 10 times smaller than in [18].

### Population profiles

For this generic population model, we point out that for costs that are not exceedingly large, any initial distribution of genotypes will quickly converge to an evolution with essentially a Hardy-Weinberg proportion—see Fig. SI1 in the S1 Text for further discussion and results. This is usually referred to as Quasi-Hardy-Weinberg Equilibrium, and it has been observed in many simpler models [51, 52], but is likely valid in more complex models as well [53, 54]. Therefore, from an initial baseline population that is entirely susceptible—given in Table SI1 in the S1 Text—we define initial conditions with the resistance allele, and that have a Hardy-Weinberg proportion. These initial conditions are classified according to the initial frequency of the resistance allele in the population, namely:

***p*-Resistant Profile (*p*-RP)** This is a population where the genotypes frequencies is (1 − *p*)^2^ for SS, 2*p*(1 − *p*) for SR and *p* ^2^ for RR.
**Mainly Resistant Profile (MRP)** This is the archetypical profile to represent a population overtaken by resistant individuals: a 0.98-RP population. It could possibly be achieved by subjecting a wild population, for which resistance costs are sufficiently small, to intense selective pressure. Indeed such a MRP scenario was reported in [34], as a result of indiscriminate application of insecticide.

### Computational implementation

The model was implemented in Octave (version 3.8.2-2) using the ode78 command from the ODE-package which implements a 7–8 Dormand-Prince method. Results were also cross-checked using the lsode command which implements an implicit Adams method. All graphics but two were produced using the package Veusz (version 1.20.1), which also provided the least-squares fit. Scilab (version 5.5.0) provided the routines for bi-cubic spline interpolation and 2-D contour computations. LaTeX scripts were employed to generate the remaining two figures.

## Results

In the absence of selective pressure of insecticide, the frequency of a costly resistance allele is expected to drop down, as indeed observed in the field [34]. Thus, any *p*-RP population will evolve towards a susceptible population. Henceforth, such movement is going to be termed *population reversal*. We identify the time of reversal as the time when the number of parous winged individuals in the population that have the resistant homozygous genotype is equal to the number of those that have the susceptible homozygous genotype. This is naturally a hallmark of reversion, since the time-scale of the evolution after this event is considerably faster. It also turns out that, for the model used, such a time is unique, even when considering seasonality. For populations with frequencies close to the Hardy-Weinberg equilibrium and consisting mainly of homozygous resistant individuals, the time to population reversal nearly coincides with the observed peak in the number of heterozygous individuals in the population, as expected.

### A first look at resistance persistence

We investigated the characteristic time-scale of resistance persistence by studying the time evolution of a MRP population, with modulating costs *C* = 0.005, 0.05, 0.15 and 0.25. The qualitative behaviour of the solution is similar for all stages, albeit with different population size scales. Further analysis was then focused on the parous winged mosquitoes as those are the individuals contributing to procreation and, in the case of female *Aedes aegypti*, to dengue virus transmission to humans.

**Fig 2:**
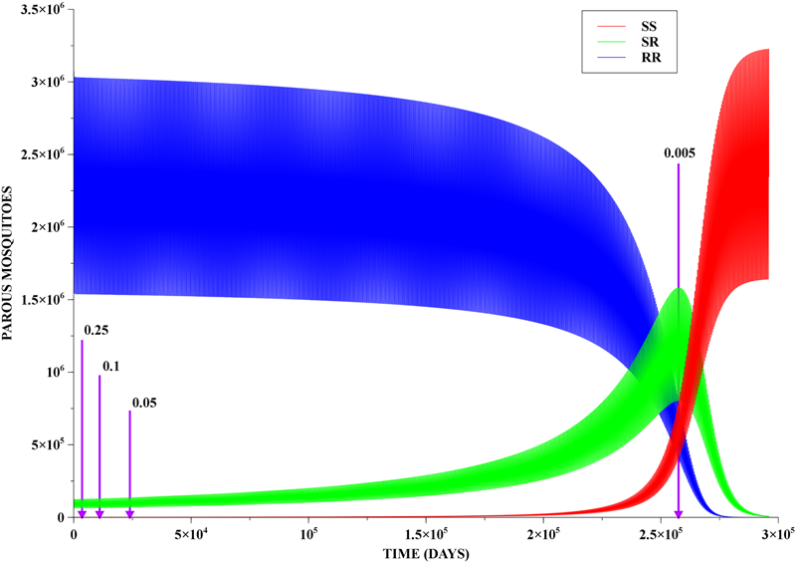
Population reversal. Evolution of a MRP population with a modulating cost of 0.005. Notice that the peak of SR is indeed very close to the reversal point, and after this peak the SR population decays very fast. In addition, we have also indicated the reversal times when the costs are 0.05, 0.1 and 0.25.

Fig 2 presents, for a modulating cost of 0.005, the time evolution of the three genotypes for 296,000 days, which is sufficiently long to allow for population reversal. Additionally, the reversal times for all the considered costs are indicated by magenta lines. Reversal times vary considerably, but even for the largest cost it is still of a significant order from the point of view of public health policies—cf. Table 2.

**Table 2:** Reversal times for a MRP population and various costs.

	Reversal Time	
Cost	(days)	(years)
0.005	257497	705
0.01	127697	350
0.05	23941	66
0.1	11064	30
0.25	3574	10

Reversal times for a MRP population obtained from the model. Notice that even for larger costs, these times are at least a decade.

**Fig 3:**
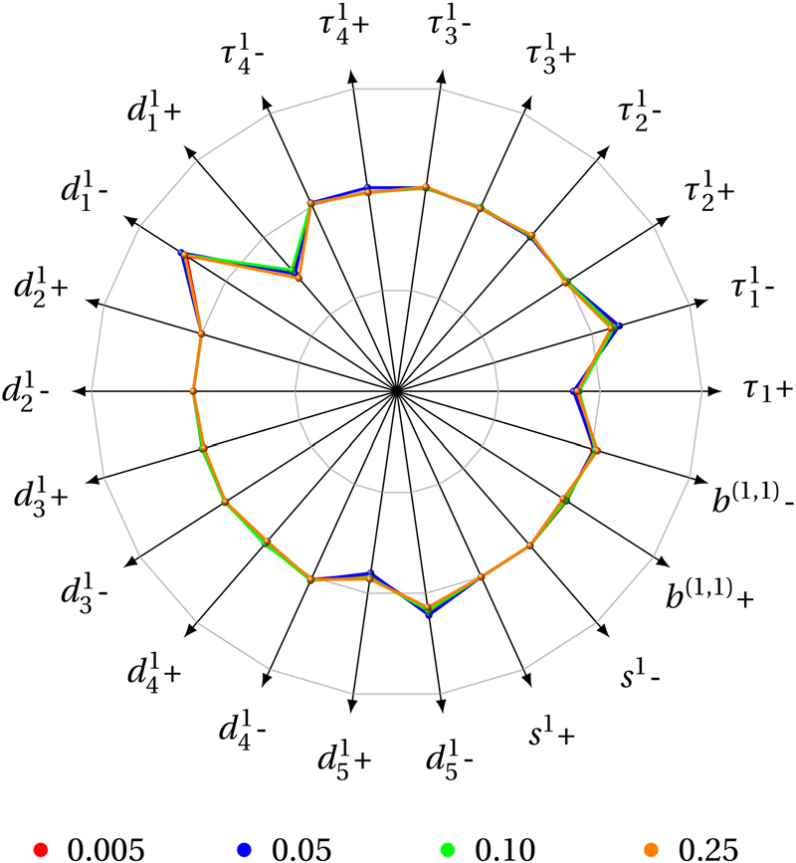
Elasticity analysis. An elasticity analysis of the reversal times, with a deviation of 1% from the corresponding values, for all the 4 costs displayed in Fig 2. The outer, intermediate, and inner circles indicate variations of 1%, 0% and -1%, respectively. The elasticities of each parameter are nearly invariant for the different costs investigated, which suggests that, as far as stability to small perturbations in the model parameters is concerned, the behaviour is uniform in cost.

We have also assessed the reliability of computed reversal times with respect to small deviations from the model parameters values used in the *in-silico* experiments, by means of a univariate elasticity analysis. This analysis indicated that the deviation in reversal time is, at the most, of the same order of the magnitude of the parameter perturbation, and hence that the model is stable regarding small uncertainties in the model parameters. Fig 3 presents a radar chart summarising the analysis.

**Fig 4:**
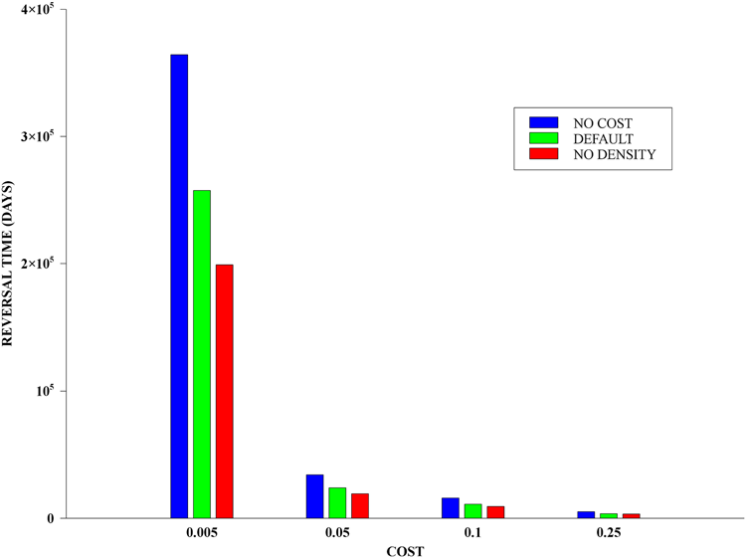
Density regulation and reversal times. Bar chart of reversal times for: 1) when density-dependent regulation is identical for all genotypes (NO COST); 2) when density-dependent is stronger for the genotypes with the resistance allele (DEFAULT); and 3) in the absence of such regulation (NO DENSITY). Notice that these times are decreasing, and that for small costs the shortest time is around 45% smaller than the longest one.

The role of intra-specific competition in the reversal times turned out to be both important and subtle. In comparison with the model used, reversal times obtained with the corresponding model without density-dependent regulation—presented in the S1 Text—were considerably shorter. On the other hand, for populations without costs imposed on the intra-specific death rate, the obtained reversal times were much longer—cf. Fig 4. In contradistinction, however, we show in the S1 Text that the baseline intra-specific death rate, *s* ^1^, does not play any role in the population dynamics, except as a scaling parameter for its total size.

### Cost and initial frequency dependence

**Fig 5:**
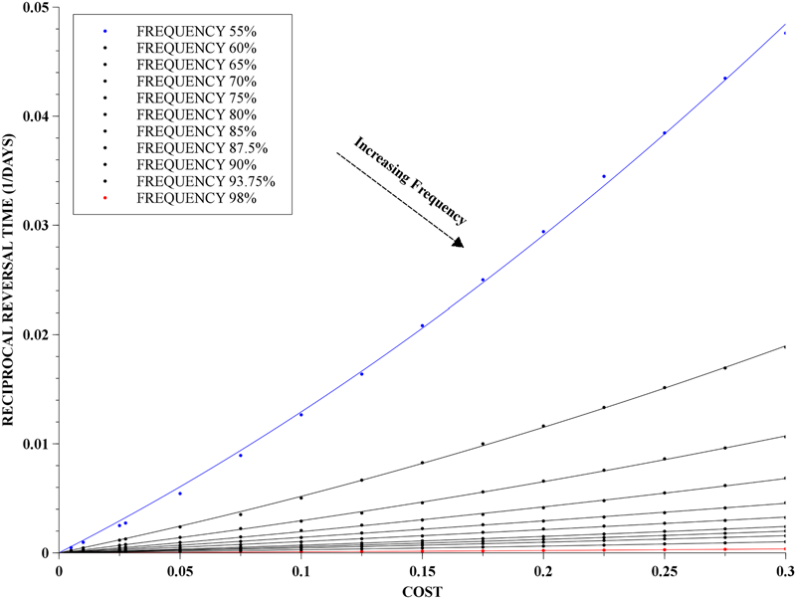
Reciprocal of reversal time as a function of cost. Dependence of the reciprocal of reversal time on the cost together with parabola least-squares fits for each set of points of the same initial frequency of the resistance allele. The costs vary from 0.005 up to 0.3 and the frequencies of the resistance allele were in the range of 55% to 98%.

The dependence of reversal times on cost and initial frequency of the resistance allele is important for two reasons: both parameters can be very much dependent on the specific population, and the range of estimates of the former is as large as controversial. For *p*-RP populations in the range of 0.55 to 0.98, *i.e.* MRP, we investigated the dependence of reversal time on cost, and this is presented in Fig 5. The range of reversal times spans a few orders of magnitude: from a few years for very high costs, up 1,000 years for very small costs. As a by-product, we were able to least-squares fit a parabola to the mean reversal rates, *i.e.* the reciprocals of reversal times, namely:

$$\begin{array}{c} {\text{Rev}}_{\text{rate}}:=\frac{1}{T_{\mathrm{reversal}}}=a_{1}C+a_{2}C^{2}, \end{array}$$ (9)

where the reversal time *T*_reversal_ is measured in days and *C* is the modulating cost as given in Eq 7, and the fitting coefficients, *a*_1_ and *a*_2_, depend on the initial frequency of the resistance allele, *p*. The fit provided by Eq (9) is very accurate, with the maximum error in the fitting ranging from 10^−6^, when *p* = 0.55, to 10^−12^ when *p* = 0.98.

**Fig 6:**
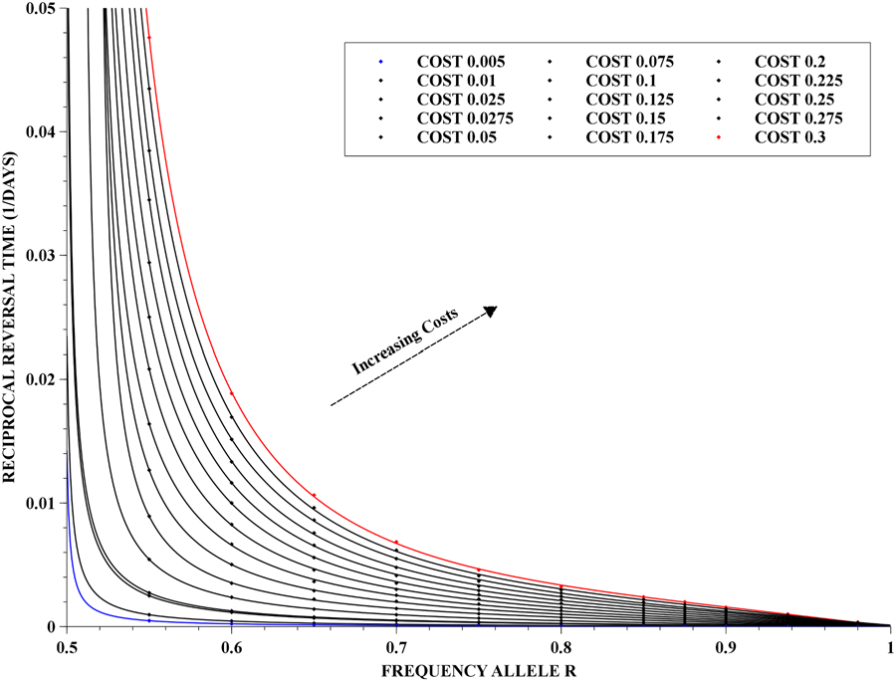
Reciprocal of reversal time as a function of initial frequency. Dependence of the reciprocal of reversal time on the initial frequency of the resistance allele together with rational least-squares fits for each set of points of the same cost. The costs vary from 0.005 up to 0.3 and the frequencies of the resistance allele were in the range of 55% to 98%.

We also present the mean reversal rates as a function of the initial frequency of the resistance allele, for fixed costs in the range of 0.005 to 0.3. These results are presented in Fig 6 together with the fit of a rational function given by:

$$\begin{array}{c} {\text{Rev}}_{\text{rate}}:=\frac{1}{T_{\mathrm{reversal}}}=\frac{a_{0}+a_{1}p+a_{2}p^{2}+a_{3}p^{3}}{p-p_{*}},\;p>p_{*}, \end{array}$$ (10)

where *p* is the initial frequency of the resistance allele, and the fitting coefficients, *a*_0_, …, *a*_3_ depend on the cost, *C*. The parameter p* is the *critical frequency* at which reversal occurs—and for a population dynamics close to a Hardy-Weinberg proportion, it is approximately 1/2. The fit provided by Eq (10) is also very accurate, with the maximum error in the fitting ranging from 10^−8^, when *C* = 0.3, to 10^−14^ when *C* = 0.005.

### Overall picture and applications

**Fig 7:**
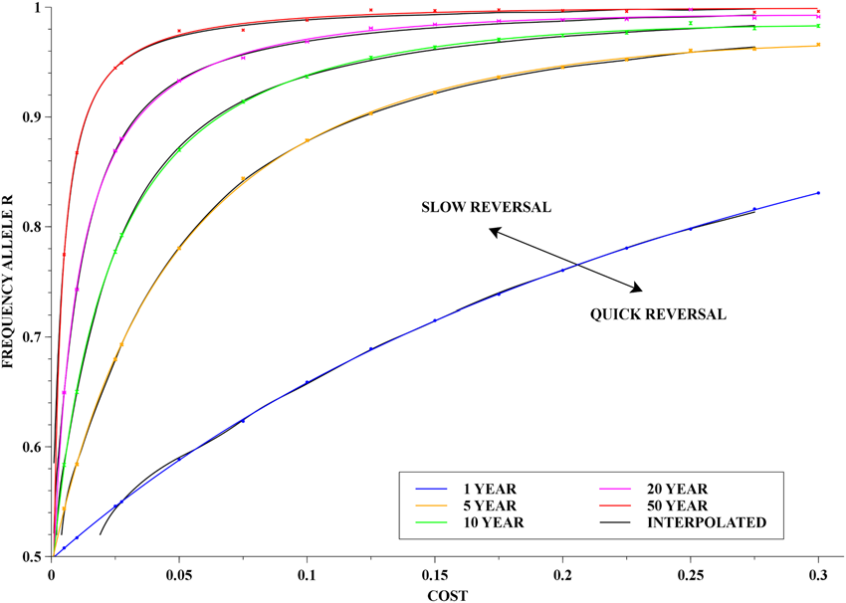
Reversal time contours. Level curves of various reversal times in the cost and initial frequency domain. The colour lines were obtained by the least-squares fitting method whilst the thin black curves were obtained from the interpolated surface—see main text for detailed explanation.

In order to get the dependence on both cost and initial frequency, one could use the fitting above, and obtain a surface for the mean rates of reversal, Rev_rate_, or for their reciprocal—the reversal times. However, it turns out that a more informative picture is to plot level curves of the reversal times. These curves were obtained in two different ways: (i) for a given reversal time, we solved (Eq 10) for p, *i.e.* the initial frequency of the resistance allele, for each one of the simulated costs. The points obtained were then further least-squares fitted to another rational function which consisted of a linear numerator and a quadratic denominator. (ii) from all the computed data, we constructed a bi-cubic spline, and then numerically solved for the level curves. The results are shown in Fig 7.

As an application we use the interpolated curves in a crude way to estimate possible reversal times for some of the field populations described in [36]. In this vein, we identified the two resistance alleles as a single resistance allele. We singled out five field population samples from four different cities: Aracaju (2010; 2012), Campo Grande (2010), Duque de Caxias (2012) and Vitória (2010). From these samples, the consolidated frequencies for the resistant genotype are 0.385, 0.433, 0.867, 0.966, and 0.833, respectively. Notice that for all these samples, the Hardy-Weinberg proportion hypothesis was accepted. Thus, the initial frequencies of the resistance allele are 0.620, 0.658, 0.931, 0.983 and 0.913, respectively.

**Fig 8:**
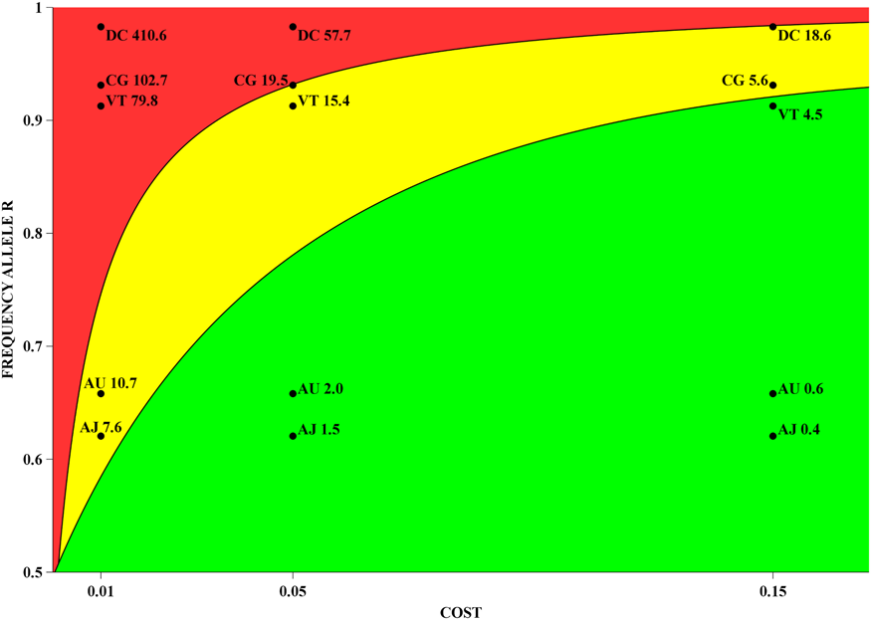
Reversal times of five field populations from four Brazilian cities. The frequencies measured in the field were plotted against three different costs: 0.01, 0.05, and 0.15. The cost-frequency space was also divided into three colour coded areas, which give an indication of the severity of resistance. The internal boundary curves correspond to reversal times of 5 and 20 years. The five populations are coded as follows: Aracaju (2010)—AJ; Aracaju (2012)—AU; Campo Grande (2010)—CG; Duque de Caxias (2012)—DC; Vitória (2010)—VT.

Using the interpolated curves, we readily find the reversal times in years for the above populations, for three different costs, namely 0.01, 0.05 and 0.15—see Table 3. We also present these reversal time estimates in graphical form in Fig 8, where the two level curves correspond to the 5 and 20 years reversal times, and are used as proposed boundaries for short, medium and long term dynamics.

**Table 3:** Reversal times for field populations.

	Reversal Time (years)		
Population	*C* = 0.01	*C* = 0.05	*C* = 0.15
Aracaju (2010)	7.6	1.5	0.4
Aracaju (2012)	10.7	2.0	0.6
Campo Grande (2010)	102.7	19.5	5.6
Duque de Caxias (2012)	410.6	57.7	18.6
Vitória (2010)	79.8	15.4	4.5

Reversal times estimated from the model using the frequency of the resistant allele in five field populations from four cities in Brazil and a choice of three representative costs (C)—see main text for detailed discussion.

### Remarks on alternative cost structures

We have also investigated the effects of different cost structures upon reversal times as compared to the default cost structure given by Eq (7).

**Fig 9:**
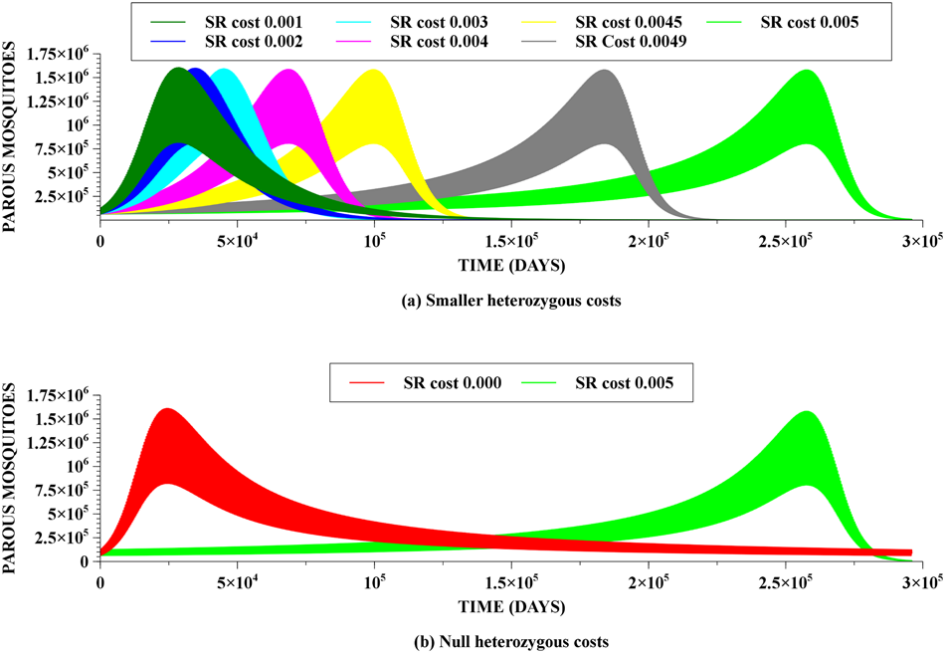
Dependence of reversal time on different costs for heterozygous individuals. Homozygous resistance cost was kept at 0.005. In 9(a) we have smaller, but non-zero heterozygous costs. Notice that reversal times are significantly smaller. In 9(b) we have null heterozygous costs. Again, there is a noticeable reduction in reversal time, but in this case notice a fatter tail for the heterozygous genotype decay.

Naturally, if fitness costs to heterozygous individuals are smaller than to the homozygous resistant individuals, then the corresponding reversal times will be shorter. Some of these results are shown in Fig 9(a). Notice that the lower are the costs impinging on the heterozygous individuals the longer these individuals remain existing after the peak. In this respect, the case of null costs for heterozygous individuals is particularly interesting, and it is shown in Fig 9(b). In this case, while there is a significant reduction on reversal time, it is still large—around eighty years—but the decay in the number of heterozygous individuals in the populations is now much slower. Indeed, while there is a considerable reduction of the resistance allele frequency after the heterozygous peak, the remaining fraction is still significant, and it persists for times even longer than in the default cost case allowing for a rapid increase in frequency should the selective pressure be resumed.

**Fig 10:**
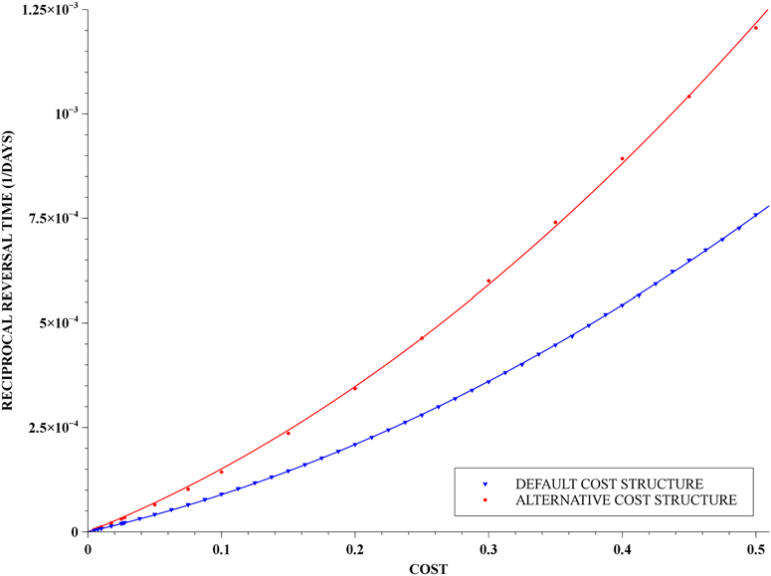
Reciprocal of reversal time as a function of cost. A parabola, shown in the lower curve in blue, was very naturally fitted to the points obtained numerically for costs impinging solely on death and oviposition rates—the default cost structure—and the least-squares fitting using Eq (9). The upper curve, in red, presents the corresponding data for costs also impinging on transition rates—the alternative cost structure. The costs vary from 0.005 up to 0.5 and the initial frequency of the resistance allele was 98% in both curves.

Also, we notice that if we allow the costs to impinge on the transition rates—namely $\text{C}(j,\tau_{i}^{1})\neq 0$ in Eq (3), for *j* = 2, 3—then we get reversal times that are shorter than those obtained with the default cost structure. This is counter intuitive since Eq (3) leads to longer transition times, and hence longer generations times. Notice, however, that in this specific case the genotypes SR and RR are contributing less to the gene pool than the SS genotype, in a given period of time, and therefore resistance loss is accelerated. Nevertheless, Fig 10 shows that for small costs, the reversal times are of the same order of magnitude than those given by the default cost structure. Furthermore, for moderate costs the reversal times are still significant within the time-scale of public health interventions: from five to twenty years.

## Discussion

We presented a comprehensive *in silico* study for a stage-structured mosquito population, with a genetically mediated resistance determined by two alleles in a single locus, in the absence of insecticide selective pressure. The present model provides an insight into the persistence of resistance behaviour of the *Aedes aegypti* mosquito population. In the absence of a more extensive knowledge of the cost impacts, we have adopted a simplified cost structure: resistance impinged a uniform modulating cost on birth and death rates on all genotypes with the resistance allele.

We then focused in understanding what we have termed the reversal time—or its reciprocal, the mean reversal rate. This quantity is meaningful for a population that has more homozygous resistant than homozygous susceptible individuals, and it is defined as the time at which the subpopulations of SS and RR genotype are of equal size. If the population dynamics approximately satisfies the Hardy-Weinberg proportion (HWP), then the reversal time can be equivalently defined as the time when both alleles have the same frequency. The reversal time yields the expected time for a wild population overtaken by a population of resistant mutant individuals to revert to susceptibility, if selection pressure is removed. The latter being less fit in natural conditions, but better fit under insecticide selection pressure.

The *in silico* experiments were performed with a variety of cost values, *C*, and initial frequency, *p*, of the resistance allele—in our terminology, this is a population with a *p*-Resistant Profile or *p*-RP. The extreme case of a 0.98-RP was termed a MRP. Populations with such an extreme resistance profile are not hypothetical and have been observed in field studies [34, 36, 43]. Overall, the experiments presented some interesting phenomena, both qualitative and quantitative: (a) for the range of costs investigated the population dynamics closely follows HWP—though they are not in equilibrium. This occurred irrespectively of the initial condition satisfying or not these proportions. In the latter case, the dynamics quickly (of the order of six months) evolved to HWP. Consequently, the reversal times very nearly coincide with the SR peak in the evolution. (b) For a MRP population, even large costs are associated to reversal times that are long from a public health point of view.

The influence of density-dependent regulation on reversal times is, in itself, an interesting issue. The results in the S1 Text show on one hand that the baseline rate is irrelevant for the population dynamics, and only acts as a scaling parameter for its total size. On the other hand, as seen in Fig 4, we have: (a) If competition is uniform, then reversal times are much longer compared to the case when both resistant genotypes have associated costs. (b) In the absence of density-dependent regulation—as is usually the case in genetic models—reversal times are shorter than when it is present. These differences are particularly acute in the small costs regime, and it indicates that density dependence indeed slows down the evolutionary dynamics. Such behaviour in ecology is well known, in the sense that elimination of parasites can be harder when there is density-dependent competition [55, 56].

We performed a systematic mapping of reversal times both as function of the cost and as function of the initial frequency of the resistance allele. The results suggested simple functional relationships, namely Eqs (9) and (10). We are not aware of any results along these directions, and while we were not able to derive them analytically, we present in the S1 Text a simplified population dynamics model for which the reversal time can be obtained explicitly. This suggests that it should be possible to obtain those relationships analytically, an issue that will be discussed elsewhere.

Moreover, Eq (10) indicates that the reversal time is a highly nonlinear function of the initial frequency of the resistance allele. This has a number of implications:

- As shown in [34, 36, 43], field populations can exhibit a large presence of the resistance allele in the population. In these cases, reversal times can be expected to be rather long, with direct implications for public health policies. Indeed, in Brazil, the Ministry of Health has suggested a modification of the insecticide substitution policy that was due to field findings indicating that populations with large frequencies of the resistance allele were taking too long to revert to a susceptible state after interruption of insecticide use—cf. [57].
- Laboratory experiments that perform competitive assays, without insecticide selective pressure, among susceptible and resistant populations usually suggest shorter reversal times [38, 58]. However, these laboratory experiments do not have very high initial frequencies of the resistance allele. Such initial frequencies will indeed lead to fast reversals, and these are not incompatible with the long reversal times observed in a MRP-like population. This raises an alert for the risk of excessively optimistic estimates obtained from those experiments.

The combined information of all *in-silico* experiments can be conveniently visualised in the 2-D plot of level curves for reversal times in cost and initial frequency parameter space—cf. Fig 7. From a practical point of view, this is probably the most useful figure, and it is more informative than a plot of the surface of reversal times.

The information contained in this 2-D plot can be used to help public health officers in assessing and managing resistance development. More precisely, we suggest the following procedure: firstly, we choose two reversal times *T*_*i*_ that define three regions that are colour-coded in green, yellow and red, and that characterise small, medium and long term reversals. Then, for a given field measure of the frequency *p* of the resistance allele, we choose a list of costs that might plausibly be imputed to the corresponding populations, and plot the points (*c*_*i*_, *p*) for *c*_*i*_ in the list. This is was done in Fig 8 for three costs, and a set of five field populations in four different cities in Brazil.

From the results in Fig 8, we observe that the four cities can be split in three scenarios: Duque de Caxias is already in a serious condition of resistance development. Campo Grande and Vitória are in an intermediate condition, and depending on the cost can also be considered in a serious condition. Aracaju is likely to have short reversal times, but its condition is worsening in time.

The actual value of the cost parameter is hard to be measured, and how the cost spreads over different life parameters is also not precisely known. Even its magnitude is a controversial matter: qualitative estimates vary from very small [30] to very large [38]; see also [29, 39]. In addition, evolution mechanisms that remove the deleterious effects, as gene duplication or compensatory mutations for instance, may cause considerable reduction of costs, and eventually lead to the fixation of the resistance genetic characteristic in the population. Indeed, especially when taking into account the mosquito’s short generation time, the use of constant costs—or frequency independent fitness—might not be entirely justifiable for larger times.

The use of alternative cost structures led to somewhat different results: when costs were allowed to impinge on transition rates, the reversal times were shorter, though still significant in the small cost regime. The case of heterozygous advantage—smaller costs for the SR genotype—significantly reduced the reversal times, with a caveat: in comparison with the default cost structure, the resistance allele persists in the population for very long times. Therefore, populations with a small cost to the heterozygous individuals would be prone to a fast return to a MRP population, should an insecticide selective pressure be applied. Indeed, the persistence of costly allele under heterozygous advantage is well known in population genetics models [52], and calls the attention to the importance of developing a better understanding of the actual cost structure of resistance.

This work can be further extended considering a variety of aspects:

- From a more theoretical perspective, it raises several questions as to a mathematical justification of the formulae obtained for the reversal times.
- It would be also interesting to explore mathematically how density dependence reduces the rate of resistance loss, and its relationship to the competitive response of the different genotypes.
- From a modelling perspective, the consideration of more detailed cost structures is a natural direction. Nevertheless, this comes with a caveat: the lack of detailed field data on resistance to indicate useful cost attributions.
- Another very natural extension is to study multi-factor models, either multi-loci or with more than two alleles, as inferred from [31–33].
- Additional extensions would include considering spatial dependence. This is motivated by results showing that intra-specific competition, which is highly localised in space, can affect life-story parameters—cf. [59–61].
- Future analysis should also study the effects of various regimens of insecticide application on population control and optimisation procedures taking into account factors affecting resistance fixation.

## Supporting Information

S1 Text Additional model details and further discussion. Oviposition function and initial condition. Hardy-Weinberg equilibrium. The model without density-dependent regulation. Scaling invariance. Exact computation of reversal times in a simple setting.
(PDF)
