# Supplementary material for: Costly Inheritance and the Persistence of Insecticide Resistance in Aedes aegypti Populations
Source: PLoS One. 2015 May 1;10(5):e0123961. doi: 10.1371/journal.pone.0123961 (PMC4416794; doi:10.1371/journal.pone.0123961)
Supplement: S1 Text — Oviposition function and initial condition. Hardy-Weinberg equilibrium. The model without density-dependent regulation. Scaling invariance. Exact computation of reversal times in a simple setting. (PDF) [file pone.0123961.s001.pdf]

Costly Inheritance and the Persistence of Insecticide Resistance in *Aedes aegypti* Populations

Helio Schechtman and Max O. Souza

Supporting Information

Additional model details

For convenience, we recall the population dynamics model for the mosquitoes described in the main text:

$$\begin{cases} \dot{Y}_{1,j} &= B(j, Y_{5,\cdot}) - \left(\tau_1^j(t) + d_1^j\right) Y_{1,j}, \\ \dot{Y}_{2,j} &= \tau_1^j(t) Y_{1,j} - \left(\tau_2^j(t) + d_2^j + s^j \sum_{l=1}^3 Y_{2,l}\right) Y_{2,j}, \\ \dot{Y}_{3,j} &= \tau_2^j(t) Y_{2,j} - \left(\tau_3^j(t) + d_3^j\right) Y_{3,j}, \\ \dot{Y}_{4,j} &= \tau_3^j(t) Y_{3,j} - \left(\tau_4^j + d_4^j\right) Y_{4,j}, \\ \dot{Y}_{5,j} &= \tau_4^j Y_{4,j} - d_5^j Y_{5,j}; \end{cases} \tag{1}$$

where t is measured in days, and $Y_{i,j}$ denotes stage i within genotype $j = 1, 2, 3$.

Also, as already discussed in the main text, we have

$$\tau_i^j(t) = \bar{\tau}_i^j \left(1 + \delta \sin\left(\frac{2\pi t}{\bar{y}} + \theta_i\right)\right), \quad i = 1, 2, 3, \quad j = 1, 2, 3; \tag{2}$$

$$\bar{\tau}_i^j = \bar{\tau}_i^1, \quad s^j = s^1 \left(1 + \mathfrak{C}(j, s^1)\right), \quad i = 1, \ldots, 4; \quad j = 1, 2, 3; \tag{3}$$

and

$$d_i^j = d_i^1 \left(1 + \mathfrak{C}(j, d_i^1)\right), \quad i = 1, \ldots, 5; \quad j = 1, 2, 3. \tag{4}$$

Oviposition function

The oviposition function $B(j, Y_{5,\cdot})$ models the production of eggs of specific genotype due to the sexual encounters of female and male parous winged mosquitoes, and are given by:

$$B(1, Y_{5,\cdot}) = \frac{Y_{5,1}^2 b^{(1,1)} + Y_{5,1} Y_{5,2} b^{(1,2)} + 0.25 Y_{5,2}^2 b^{(2,2)}}{Y_{5,1} + Y_{5,2} + Y_{5,3}}, \tag{5}$$

$$B(2, Y_{5,\cdot}) = \frac{Y_{5,1} Y_{5,2} b^{(1,2)} + 0.5 Y_{5,2}^2 b^{(2,2)} + Y_{5,2} Y_{5,3} b^{(2,3)} + 2 Y_{5,1} Y_{5,3} b^{(1,3)}}{Y_{5,1} + Y_{5,2} + Y_{5,3}}, \tag{6}$$

$$B(3, Y_{5,\cdot}) = \frac{Y_{5,3}^2 b^{(3,1)} + Y_{5,3} Y_{5,2} b^{(3,2)} + 0.25 Y_{5,2}^2 b^{(2,2)}}{Y_{5,1} + Y_{5,2} + Y_{5,3}}; \tag{7}$$

with

$$b^{(j,j)} = b^{(1,1)} (1 - \mathfrak{C}(j, b^{(1,1)})), \quad j = 2, 3; \tag{8}$$

and

$$b^{(m,n)} = \sqrt{b^{(m,m)} b^{(n,n)}}, \quad m, n = 1, \ldots, 3, \quad m \neq n. \tag{9}$$

An important feature of this oviposition function is that it is homogeneous of degree one, namely, if $\lambda > 0$ then

$$B(j, \lambda Y_{5,\cdot}) = \lambda B(j, Y_{5,\cdot}). \tag{10}$$

Initial conditions

Extensive numerical experimentation with System (1) shows that for the parameters considered, and various initial conditions without heterozygous or homozygous resistant genotypes, the solution converges to a unique periodical solution, as expected.

For the sake of reproducibility of the results, we fix an arbitrary initial condition, namely:

$$\begin{aligned}
&Y_{1,1}(0) = 0.979N,\\
&Y_{2,1}(0) = 0.006N,\\
&Y_{3,1}(0) = 0.001N,\\
&Y_{4,1}(0) = Y_{5,1}(0) = 0.007N,\\
&N = 4 \times 10^7;
\end{aligned}$$

and then we evolve it for a long period. This procedure yields the basic initial condition (BIC) described in Table SI1.

Quantity	Value	Fraction of Total	Description
$Y_{1,1}(0)$	469,300,000	0.9870	Initial total number of eggs
$Y_{2,1}(0)$	2,101,800	0.0044	Initial total number of larvae
$Y_{3,1}(0)$	343,790	0.0007	Initial total number of pupae
$Y_{4,1}(0)$	934,610	0.0020	Initial total number of non-parous winged mosquitoes
$Y_{5,1}(0)$	2,823,400	0.0060	Initial total number of parous winged mosquitoes

Table SI1: Basic initial condition with a pure susceptible population obtained after evolution through 4,384 days or 12 years and 1 day.

This initial condition is then perturbed by redistributing this purely susceptible population into the three genotypes to generate the basic population profiles p-RP (and in particular MRP) as described in the subsection **Population profiles** in the main text.

As expected, the long-term behaviour of the solutions for these population profiles or perturbations of them were similar: they all converged to the unique periodic solution that originated the BIC.

Hardy-Weinberg equilibrium

Under non-preferential mating, and for simplified models, it is known that a slight departure of the condition of absence of genotype advantage—i.e. absence of selection—leads, very quickly, to a quasi-Hardy-Weinberg equilibrium [1, 2]. This was also verified for the model used in the experiments, provided the associated fitness costs are not too excessive. In Figure SI1, we show a ternary plot (or De-Finetti diagram) [3] of the evolution. Significant deviations from HW equilibrium, shown as the magenta line, start to appear only for costs greater than 0.2.

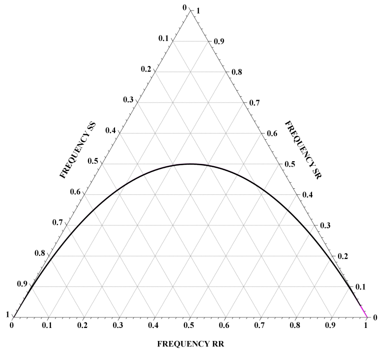

(a)

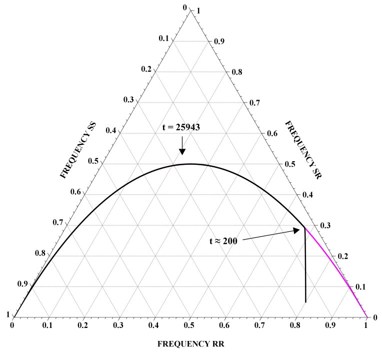

(b)

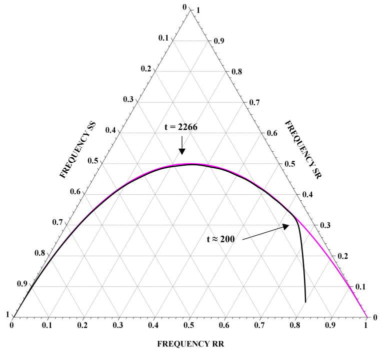

(c)

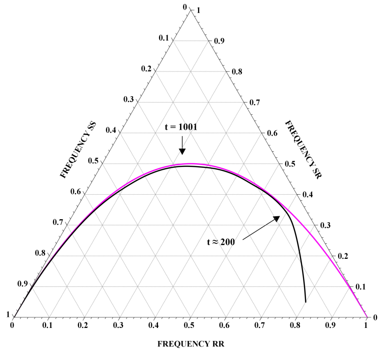

(d)

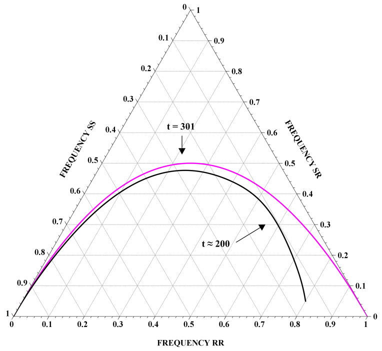

(e)

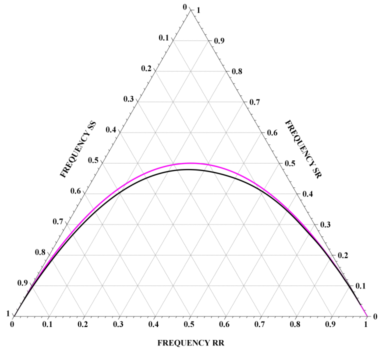

(f)

Figure SI1: **Quasi-Hardy-Weinberg Equilibrium.** Ternary plots for various cost impinging on a non Hardy-Weinberg initial population and a quasi-HW MRP population. The initial condition for the non HW population is given by 0.8 RR, 0.05 SR and 0.15 SS—which does not satisfy the HWP. The corresponding costs are 0.005 in SI1a and SI1b, 0.05 in SI1c, 0.10 in SI1d, 0.25 in SI1e and SI1f.

The model without density-dependent regulation

When studying population genetics, in general, models do not have regulated populations and, indeed, the population will grow exponentially large. In the model described in the main text, this corresponds to assume $s^j = 0,\ j = 1, 2, 3$. To study this simplified model it is convenient to switch to a frequency description of the population. Let

$$Z_{i,j} = \frac{Y_{i,j}}{N}, \quad N = \sum_{i=1}^{5}\sum_{j=1}^{3} Y_{i,j}. \tag{11}$$

Then $Z_{i,j}$ satisfies the following system

$$\begin{cases} \dot{Z}_{1,j} &= B(j, Z_{5,\cdot}) - \left(\tau_1^j(t) + d_1^j + \Gamma\right) Z_{1,j}, \\ \dot{Z}_{2,j} &= \tau_1^j(t) Z_{1,j} - \left(\tau_2^j(t) + d_2^j + \Gamma\right) Z_{2,j}, \\ \dot{Z}_{3,j} &= \tau_2^j(t) Z_{2,j} - \left(\tau_3^j(t) + d_3^j + \Gamma\right) Z_{3,j}, \\ \dot{Z}_{4,j} &= \tau_3^j(t) Z_{3,j} - \left(\tau_4^j + d_4^j + \Gamma\right) Z_{4,j}, \\ \dot{Z}_{5,j} &= \tau_4^j Z_{4,j} - \left(d_5^j + \Gamma\right) Z_{5,j}; \end{cases} \tag{12}$$

where

$$\Gamma = \sum_{j=1}^{3} \left[B(j, Z_{5,\cdot}) - d_1^j Z_{1,j} - d_2^j Z_{2,j} - d_3^j Z_{3,j} - d_4^j Z_{4,j} - d_5^j Z_{5,j}\right]. \tag{13}$$

Scaling invariance

In population dynamics, density dependent death usually plays the role of a scaling parameter for the carrying capacity of the associated population. In this case, it is possible to have it rescaled out of the model. For System (1), we shall prove below that this turns out to be correct for the baseline intra-specific death rate, s^1.

In order to see that System (1) has a natural scaling invariance we proceed as follows: write $\mathcal{Y}_j = (Y_{1,j}, \ldots, Y_{5,j})$. Then (1) has a vector representation given by

$$\dot{\mathcal{Y}}_j = A_j(t)\mathcal{Y}_j + F_j(\mathcal{Y}_1, \mathcal{Y}_2, \mathcal{Y}_3), \quad j = 1, 2, 3, \tag{14}$$

with

$$F_j(\mathcal{Y}_1, \mathcal{Y}_2, \mathcal{Y}_3) = (B(j, Y_{5,\cdot}), -s^1\left(1 + \mathfrak{C}(j, s^1)\right) \sum_{l=1}^{3} Y_{2,l} Y_{2,j}, 0, 0, 0)^t, \tag{15}$$

where the superscript t means transpose.

Write

$$\mathcal{Y}_j = \frac{1}{s^1}\mathcal{Z}_j, \tag{16}$$

and notice, from the homogeneous property of B as discussed in the above section **Additional model details**, that we can write:

$$F_j\left(\frac{\mathcal{Z}_1}{s^1},\frac{\mathcal{Z}_2}{s^1},\frac{\mathcal{Z}_3}{s^1}\right)=\frac{1}{s^1}(B(j,Z_{5,\cdot}),-\left(1+\mathfrak{C}(j,s^1)\right)\sum_{l=1}^{3}Z_{2,l}Z_{2,j},0,0,0)^t$$
$$=\frac{1}{s^1}G_j(\mathcal{Z}_1,\mathcal{Z}_2,\mathcal{Z}_3). \tag{17}$$

Hence, Equation (14) can be rewritten as

$$\frac{1}{s^1}\dot{\mathcal{Z}}_j=\frac{1}{s^1}A_j(t)\mathcal{Z}_j+\frac{1}{s^1}G_j(\mathcal{Z}_1,\mathcal{Z}_2,\mathcal{Z}_3),\quad j=1,2,3. \tag{18}$$

Therefore, we have

$$\dot{\mathcal{Z}}_j=A_j(t)\mathcal{Z}_j+G_j(\mathcal{Z}_1,\mathcal{Z}_2,\mathcal{Z}_3),\quad j=1,2,3. \tag{19}$$

System (19) does not depend on s^1, and hence the solutions for the original System (1) can be obtained by simply rescaling the solutions of System (19). In the case when $\mathfrak{C}(j,s^1)=0$, *i.e.* when density dependent regulation is the same for all genotypes, then the strength of such dependence can be completely scaled-out of the problem.

Exact computation of reversal times in a simple setting

We want to provide a simple example, where reversal times can be computed analytically, which suggests that some of the features of reversal times that we found in the *in silico* experiments are likely to be universal.

We consider the simplest case of the replicator dynamics with constant fitness difference, and negative selection, and assume that this difference is smooth as a function of a cost C, which vanishes for $C=0$:

$$\frac{\mathrm{d}x}{\mathrm{d}t}=-\gamma(C)x(1-x). \tag{20}$$

For Equation (20) we have that $x=0,1$ are equilibrium points, with $x=0$ being the unique element in ω-limit set.

For a given initial condition $x_0>1/2$, a similar definition of reversal time would be to look for the unique time t where $x(t)=1/2$. This can be computed analytically for this model, since it is equivalent to

$$\frac{\mathrm{d}t}{\mathrm{d}x}=\frac{-1}{\gamma(C)}\frac{1}{x(1-x)}, \tag{21}$$

which can be integrated from some initial condition $x_0>1/2$ to yield

$$t(x)=\frac{1}{\gamma(C)}\log\left(\frac{(1-x)x_0}{x(1-x_0)}\right). \tag{22}$$

Notice that $t(x)$ is then the time elapsed by the solution with an initial condition x_0 and it has now the value of x. Therefore, the reversal time for an initial presence x_0 can then be defined as $t(1/2)$. In this case, we then have

$$T_{\mathrm{Rev}}(x_0)=t(1/2)=\frac{1}{\gamma(C)}\log\left(\frac{x_0}{1-x_0}\right). \tag{23}$$

Hence the reversal rate is

$$R_{\mathrm{Rev}}(x_0)=\gamma(C)\left[\log\left(\frac{x_0}{1-x_0}\right)\right]^{-1}. \tag{24}$$

For fixed x_0, Equation (24) can be expanded in powers of C, and this is precisely the kind of the representation described by Equation (9) in the main text. In addition, for a fixed value of C, we have

$$\lim_{x_0 \to 1/2^+} R_{\mathrm{Rev}}(x_0) = +\infty \quad \text{and} \quad \lim_{x_0 \to 1^-} R_{\mathrm{Rev}}(x_0) = 0.$$

This is consistent with the representation given by Equation (10) in the main text. Furthermore, it can be checked that $R_{\mathrm{Rev}}(x_0)$ is convex so that its graphic is qualitatively similar to the curves obtained in Figure 6 in the main text. Nevertheless, in spite of all these similarities, the simulation data could not be fitted to such a representation.

References

1. Hoppensteadt F. Mathematical theories of populations: demographics, genetics and epidemics. Society for Industrial and Applied Mathematics, Philadelphia, Pa.; 1975. Regional Conference Series in Applied Mathematics.

2. Ewens WJ. Mathematical Population Genetics 1: I. Theoretical Introduction. Springer Science & Business Media; 2004.

3. Graffelman J, Camarena JM. Graphical Tests for Hardy-Weinberg Equilibrium Based on the Ternary Plot. Human Heredity. 2008;65(2):77–84. doi:10.1159/000108939.
